# The Myth of Effortless Clinical Documentation Automation: A Critical Sociotechnical Commentary on Ambient Digital Scribes

**DOI:** 10.2196/109717

**Published:** 2026-11-11

**Authors:** Kate Preston, Ibrahim Habli, Tom Lawton, Yasmeen Hayat, Alice Cunningham, Mark Sujan

**Affiliations:** 1 Centre for Assuring Autonomy, University of York, Heslington, York YO10 5DD, York, England, United Kingdom, 44 01904 320000; 2 Bradford Teaching Hospitals NHS Foundation Trust, Bradford, England, United Kingdom; 3 Improvement Academy, Bradford, United Kingdom

**Keywords:** ambient digital scribes, clinical documentation, human factors, sociotechnical systems, artificial intelligence, AI

## Abstract

Ambient digital scribes (ADSs) are promoted to reduce clinical documentation burden, improve efficiency, and allow clinicians to focus on patients. This viewpoint examines the sociotechnical assumptions underlying these claims and argues that safe integration of ADSs requires consideration of how documentation work and clinician oversight are transformed within the wider clinical work system. Their appeal rests on 2 reassuring assumptions: that documentation work can be cleanly automated and that residual risks can be controlled because clinicians remain responsible for reviewing and correcting the output. This viewpoint argues that both assumptions can be challenged from a sociotechnical perspective. Automating the draft note does not eliminate documentation work. It transforms and redistributes it, moving clinicians from composing notes to evaluating, correcting, and editing AI-generated accounts, potentially transferring additional verification and reconciliation work to other people and later stages of care. Clinician oversight should not be treated as an automatic safety backstop. Plausible outputs may encourage cursory review, while omissions and subtle shifts in meaning can be difficult to detect. Time pressure, perceived system reliability, and organizational expectations for productivity may further weaken scrutiny. ADSs should not be designed and implemented through simple substitution of AI for documentation tasks followed by reliance on human checking. Safe integration requires sociotechnical design at the level of the wider work system, including deliberate choices about task design, organizational arrangements, and organizational learning. Participatory and context-sensitive sociotechnical design can inform explicitly what an ADS should and should not do, how the resulting documentation work should be distributed, and how impacts will be managed across the wider care system.

## Introduction

Ambient digital scribes (ADSs), a particular application of a broader range of ambient voice technology, are generative AI–enabled systems that capture clinical encounters and produce draft clinical documentation for clinician review [1,2]. ADSs can vary in their functionality and degree of integration within existing clinical workflows, with some being a separate platform or app that requires generated documentation to then be transferred manually into the electronic health record (EHR), while others are integrated directly within the EHR [3]. They are promoted as a solution to the administrative burden, dissatisfaction, and burnout that clinicians experience as a result of administrative and data entry tasks, and they come with the prospect of returning clinician time and attention to patient care [4,5]. The apparent appeal of this proposition depends on two reassuring assumptions: (1) that documentation work can be cleanly automated and (2) that any residual risks can be controlled because clinicians remain responsible for reviewing and correcting the output. Both assumptions can be challenged by analyzing the sociotechnical factors affecting work and safety. This sociotechnical analysis can be conducted through the application of approaches, models, and methods related to the discipline of human factors and ergonomics. This discipline aims to optimize work system performance and improve human well-being by taking a systems and human-centric design approach [6]. A sociotechnical analysis considers the interactions among the different elements of the work system, including people, tasks, tools, and technologies; the physical environment; and organizational factors, as well as the wider external environment in which work takes place. Sociotechnical approaches such as the systems engineering initiative for patient safety framework and the sociotechnical model for health IT by Sittig and Singh [7] can provide structured ways to examine these interactions and how they may influence performance and safety [7-11]. Such approaches have an established role in the assessment and design of health care–related technologies to understand how they will interact with existing clinical workflows and organizational contexts rather than assessing them in isolation [12].

In this context, the first assumption overlooks how introducing ADSs could transform and redistribute documentation work by creating new tasks for clinicians and potentially transferring additional verification and reconciliation work to other people and later stages of care. The second assumption treats clinician oversight as an inherent safeguard rather than something that depends on how the wider work system is designed. Therefore, this viewpoint draws on emerging empirical evidence and established sociotechnical perspectives to critically examine the assumptions underlying the use of ADSs to reduce clinical documentation burden and improve efficiency. It then considers what these assumptions mean for the safe design and implementation of ADSs. Specifically, we consider how ADSs may transform and redistribute documentation work, examine why clinician review should not be assumed to provide a reliable risk control, and explore what these issues mean for sociotechnical design and implementation across the wider clinical work system. In doing so, this viewpoint aims to support clinicians, technology developers, and health care organizations involved in the design and implementation of ADSs to recognize and address the sociotechnical conditions that shape their safe and effective use.

## Clinical Documentation Burden and the Promise of ADSs

Clinical documentation places a substantial burden on clinicians, often extending beyond the working day and reducing the time and attention available for patients. Early studies of ADSs have reported that clinicians using ADSs may spend less time updating patient notes in the EHR, with interview studies also reporting perceived reductions in workload [13,14]. Furthermore, 2 quality improvement studies suggested that integrating this technology may potentially reduce the need for completing administrative tasks out of hours, potentially improving work-life balance [15,16]. ADSs may also allow clinicians to dedicate more attention to the patient during the consultation, with potential benefits for the quality of patient-clinician interaction [4].

These early promising findings have contributed to rapid enthusiasm for ADSs. Their development and uptake have been facilitated by the growing accessibility of generative AI and by a perception that clinical documentation represents a low-risk application, with ambient voice technology typically not qualifying as a medical device under UK regulations [1]. A growing number of ADSs are now available on the market, with 24 suppliers of ambient voice technology products being listed on the National Health Service self-certified supplier registry [14,17]. There is also increasing demand from clinicians for ADS tools, with research highlighting that clinicians may be adopting such tools independently without any oversight from the wider organization [18].

The perceived need for ADSs may, in part, stem from long-standing challenges associated with EHRs and their contribution to clinician stress and burnout [19]. EHRs were initially introduced with the aim of improving the quality, efficiency, and safety of health care. However, their implementation has also been associated with unintended consequences, including changes in patient-clinician interactions and increased demands associated with data entry and documentation [19]. These challenges and the associated burden experienced by clinicians may be partly attributable to poor EHR usability, which can arise when human-centered considerations are not sufficiently incorporated into the design and development of these systems [20].

Overall, ADSs offer a compelling response to a genuine and consequential problem. However, reducing time to produce an initial draft note may not necessarily translate into a reduction in documentation work overall. Whether ADSs deliver meaningful reduction in documentation and administrative burden may therefore depend on the new work and dependencies created around their use and how these are designed within the wider clinical work system.

## Automating the Draft Does Not Eliminate Documentation Work

ADSs can produce draft clinical notes from a consultation, potentially allowing clinicians to focus on the patient rather than on note-taking [21]. However, shifting the initial production of the clinical note from the clinician to the ADS does not remove documentation work altogether. Instead, it redistributes it and creates new forms of work, moving clinicians from writing the note to reviewing and editing an AI-generated output [4,22,23].

Previous articles and features focusing on ADSs have identified several kinds of errors and potential ambiguities, including hallucinations, misinterpretations, omissions, and subtle shifts in meaning or emphasis [22,24,25]. Some errors, especially conspicuous hallucinations, might be easier to detect and remedy, but others such as omissions and shifts in interpretation might appear plausible and could be harder to identify [26]. ADSs may also struggle with contextual information that is not explicitly articulated (eg, nonverbal communication) [27]. A professionally written and apparently plausible-looking clinical note may therefore still be incomplete or misleading when used as the basis for subsequent clinical work and decision-making by a range of different health care professionals.

Evaluations of ambient AI scribes suggest that they can generate good-quality notes but that omissions and structural errors persist and require clinician review and editing [28]. There is evidence from clinicians’ explanations of the changes they make to AI-drafted notes further suggesting that editing is not a minor residual task. It involves clinical judgment about accuracy, completeness, emphasis, and whether the resulting record adequately represents the consultation [29]. Automating the first draft could therefore transform documentation from a task of composition into one of evaluating, correcting, and sometimes reconstructing an AI-generated account.

An important design consideration concerns when checking takes place. It is conceivable that the checking task cannot be done during the consultation as the digital scribe has not produced its summary yet and the purpose of using ADSs is to enable clinicians to focus on the patient. In these cases, review would take place afterward and would need to rely on the clinician remembering what was said and intended during the conversation. This may be especially problematic in busy settings involving multiple similar consultations or where checking is delayed until later in the working day. As a result, ADSs may consequently redistribute documentation work not only from composing to reviewing but also from the consultation itself to a later point in the workflow.

The work created by ADSs may also be displaced downstream to people who were not present during the consultation. Clinical notes support numerous subsequent activities, including but not limited to referrals, scheduling, patient correspondence, handover, and future clinical decision-making. Although administrative staff and clinicians already often resolve ambiguities in clinical records, ADSs may change their form and detectability, thereby creating additional workload. For example, generative AI could potentially inadvertently convert tentative discussion into apparently definite actions or blur distinctions between what was considered and what was agreed, producing inconsistencies between the narrative summary and clinical orders or structured EHR fields. It is therefore plausible that downstream users may need to reconstruct meaning or reconcile discrepancies without access to the original consultation, transferring verification work to those least able to determine what was actually said or intended.

Clinical documentation may also serve an important cognitive and educational function beyond communicating information to others. The process of constructing a clinical note can form part of clinical decision-making itself as clinicians use the emerging record to organize information, develop diagnostic reasoning, and record decisions and care plans [27,30]. This reflects the concept of external cognition, in which thinking is understood as involving interaction with external representations rather than occurring solely within the individual [31]. For medical students and resident physicians, note writing also provides opportunities to make explicit clinical reasoning [30]. If ADSs increasingly generate the initial clinical narrative, trainees may have few opportunities to engage directly in the processes of prioritizing information, synthesizing findings, and articulating diagnostic reasoning and care plans. This does not mean that ADSs are inherently detrimental to medical education, but it suggests a need for deliberate educational guardrails to preserve opportunities for independent reasoning and feedback [32].

It follows that ADSs redistribute documentation work across tasks, time, and people, often into forms that may be less visible but cognitively demanding and critical to patient safety. Realizing any efficiency benefit may depend on whether this new work is recognized and deliberately designed into clinical workflows. Measures of reduced note-taking time and faster draft production cannot, on their own, establish whether documentation work has been reduced overall [33].

Evaluation of ADSs should therefore extend beyond consideration of the time clinicians spend producing and reviewing documentation [33]. It should also account for the time and cognitive effort required for newly created tasks, including reviewing and editing AI-generated documentation. Recent work has similarly argued that evaluation should consider how ADSs reshape clinical workflows, documentation practices, and organizational processes [27]. Evaluation should also extend further downstream once the resulting record becomes part of the health system infrastructure and is used by other clinicians and staff to support communication and coordination [34]. This wider perspective is needed to determine whether apparent efficiencies with initial documentation production translate into meaningful benefits across the work system and whether unintended consequences emerge elsewhere.

## Clinician Oversight Is a Fragile Safeguard

A common reassurance surrounding ADSs and their perceived low-risk application is that the clinician remains responsible for reviewing and correcting the generated summary or note. However, formal responsibility should not be confused with effective oversight and risk control. In fact, clinicians might be held accountable for outputs they did not fully produce while being expected to detect omissions and changes in intent or emphasis that are not always readily apparent from the generated text [35]. Assertions of “human in the loop” do not, by themselves, establish that the risks associated with ADSs are adequately controlled.

Established human factors and ergonomics concepts such as automation bias and automation-induced complacency remain valuable in this context [36]. There is research suggesting that clinician performance can be degraded when automated or AI-generated outputs are incorrect, including under single-task diagnostic conditions and in medical image interpretation [37,38]. Following on from this, reviewing an ADS-generated note may present an especially difficult form of oversight task because the output is likely to be seen as broadly accurate. Depending on factors such as task complexity, time pressure, and perceived ADS reliability, AI-generated notes may be routinely experienced as “good enough,” and review may become increasingly cursory. This does not mean that clinician review will be ineffective in all circumstances, but successful review in some or even many cases does not establish clinician review as a dependable risk control. Rather, its effectiveness is likely to depend on the sociotechnical conditions in which review takes place, including clinicians’ contextual knowledge and experience, the time and information available, and the design of the surrounding workflow and technology.

The sociotechnical context might create conditions that either support or undermine effective review. Clinicians must sustain attention while reconstructing a consultation that has already concluded, often alongside competing demands and after several similar encounters. At the same time, ADSs are introduced with the promise of reducing documentation time. This may create pressure and an organizational expectation, even if implicit, on clinicians to complete the checking quickly, which might prompt rapid or superficial review. The perceived efficiency of AI note production could, therefore, create the conditions in which the output receives less scrutiny because time-consuming review may come to be regarded as unnecessary friction in a workflow designed around rapid completion.

Research across industries suggests that neither training nor accountability appear to be viable strategies for addressing automation bias and overreliance [38]. Accordingly, telling clinicians that they must check the output does not address whether they have the contextual and organizational support needed to do so meaningfully, nor should assigning responsibility compensate for system designs that could make reviewing outputs difficult or encourage rapid acceptance. From a human factors perspective, clinician oversight should be treated as a substantive risk control function whose effectiveness depends on the surrounding work system. Meaningful oversight must therefore be deliberately enabled through the design of interactions across different work system elements, including the clinical setting, tasks, interfaces, and organizational resources, rather than assumed to follow from the clinician’s continued presence.

## ADSs Require Sociotechnical Design, Not Simple Substitution

Time-saving documentation and meaningful clinician oversight should not be treated as inherent properties of ADSs. It is more fruitful to consider these as arising from interactions between the technology and the wider sociotechnical work system within which it is introduced. The appropriate unit of design, therefore, may not simply be the ADS or its user interface but the work system through which clinical encounters are converted into documented records, including their review and subsequent use. As a result, design should begin by understanding this work system and examining how ADSs change tasks and responsibilities rather than assuming that existing structures will remain stable after the introduction of technology.

This also requires moving beyond simple substitution of AI for previous human activity [39-41]. The relevant design question is not only which parts of documentation can be automated but also which combinations of clinician and ADS activity genuinely support better clinical work. Full-note generation followed by retrospective clinician review should not be accepted as the inevitable design model. Alternative forms of augmentation may be more appropriate in some settings, including systems that preserve direct clinician authorship of clinically significant judgments or help clinicians structure and recall information without attempting to produce a complete account autonomously. The allocation of work between clinician and technology should be treated as a design choice that requires evaluation rather than being based on static and decontextualized assumptions about what technology is better at and what people are better at [41].

Where organizations choose to rely on clinicians to review AI-generated notes as risk control, this review must be designed as a substantive safety-critical task. Organizations need to determine when it occurs; how much time is available; what information clinicians can use to assess the output; and how discrepancies among the note, clinical orders, and structured EHR information are resolved. This needs to be supported by appropriate interfaces and workflows, with a focus on organizational learning that can identify gaps between work as imagined and work as done in a nonjudgmental and improvement-focused way [42]. This also needs to consider that training and professional accountability may have a role, but they cannot compensate for work systems that make meaningful oversight difficult.

Work system design extends beyond clinicians reviewing ADS outputs to include how patients are informed about and consent to ADS use. Patients need clear information and transparency regarding their privacy, enabling them to give informed and meaningful consent [22,43]. This is particularly important for sensitive conversations, where patients may be less comfortable discussing issues such as substance abuse or counseling if the consultation is recorded by an ADS [44]. ADS use may therefore influence patient behavior during the consultation, including willingness to disclose sensitive information [44]. Practical design considerations include who seeks consent; how this is incorporated into the consultation; how the recording is started, paused and restarted, and stopped; and what alternative process is followed when a patient declines ADS use.

Progress in the sociotechnical design of ADSs requires participatory and context-sensitive work across different communities that extend beyond technology developers and clinicians [45]. Patient ergonomics and patient partnership approaches can help ensure that patients and families shape what safe and meaningful ADS adoption might entail [46]. Embedded human factors professionals can also support this process by applying sociotechnical and systems approaches to examine how ADSs interact with clinical workflows, roles, tasks, technologies, and organizational processes. This can help understand how changes in one element may impact others and support engagement with clinicians and patients to understand how work is done, and how ADSs may change these processes. More broadly, human factors professionals can help translate among clinical practice, technology development, implementation, and organizational learning [47].

## Conclusions

Claims that ADSs can straightforwardly improve efficiency and reduce documentation burden, with clinician review providing an effective safety backstop, are overly reductionist and potentially misleading. Automating the generation of a draft note could transform and redistribute documentation work and may transfer uncertainty to other people and later stages of care. Clinician review may form part of oversight, but it should not be treated as a sufficient safeguard. Participatory and context-sensitive sociotechnical design can inform explicitly what an ADS should and should not do, how the resulting documentation work should be distributed, and how impacts will be managed across the wider care system.

## Abbreviations

ADS: ambient digital scribe
EHR: electronic health record
